# Protective effects of celecoxib on ischemia reperfusion–induced acute kidney injury: comparing between male and female rats

**DOI:** 10.22038/ijbms.2018.29644.7156

**Published:** 2019-01

**Authors:** Farzaneh Kianian, Behjat Seifi, Mehri Kadkhodaee, Abdullah Sajedizadeh, Parisa Ahghari

**Affiliations:** 1 Department of Physiology, School of Medicine, Tehran University of Medical Sciences, Tehran, Iran; 2 Department of Physiology, School of Medicine, Hamedan University of Medical Sciences, Hamedan, Iran

**Keywords:** Acute kidney injury, Celecoxib, Gender difference, Oxidative stress, Reperfusion injury

## Abstract

**Objective(s):**

There is increasing evidence for the importance of gender in different diseases; however, the role of gender in response to treatments is still unknown. Therefore, this study investigated the impact of gender on the protective effects of celecoxib in ischemia reperfusion (IR)-induced acute kidney injury.

**Materials and Methods:**

In this experimental study, rats were randomly divided into 6 groups (n=6): IR, sham and celecoxib groups of males and females. In IR groups, after orally receiving saline for 5 days, renal pedicles were clamped for 55 min and then kidneys were reperfused for 24 hr. In the sham groups, clamping of renal pedicles was not performed. In the celecoxib groups, 30 mg/kg celecoxib was given orally for 5 days before induction of ischemia. Plasma was collected to determine creatinine (Cr) and blood urea nitrogen (BUN). Kidney tissue samples were also stored for examining the histopathology and measuring malondialdehyde (MDA) levels and superoxide dismutase (SOD) activities.

**Results:**

IR caused significant increases in plasma Cr (*P<*0.05), BUN (*P<*0.05) and renal histopathological damages in both genders. Also, induction of IR resulted in significant increase of MDA levels (*P<*0.05) and decrease of SOD activities (*P<*0.05) in the kidney in both genders. Celecoxib administration prevented the IR-induced functional, histopathological and oxidative changes in both genders by similar degrees.

**Conclusion:**

This study suggested that in similar pathological conditions, celecoxib improves renal function and histopathological damages and attenuates oxidative stress in both genders by the same degrees. These protective effects of celecoxib on IR-induced kidney injury are gender-independent.

## Introduction

Acute kidney injury (AKI) is a common clinical disorder that is associated with significant morbidity and mortality (1). It is characterized by abrupt (within hr) decrease of function and structural damage to the kidneys (2).

Renal ischemia reperfusion injury (IRI) is one of the main causes of AKI, which is related to different clinical situations such as shock, low cardiac output and kidney transplantations (3). In these conditions, the restoration of blood flow after a period of deprived circulation leads to renal damages (4).

Oxidative stress, an imbalance between oxidant (e.g. reactive oxygen species, ROS) and anti-oxidant (e.g. superoxide dismutase, SOD) systems, is implicated in the pathophysiology of renal IRI and consequent AKI (5). One of the reactions involved in the oxidative stress process is initiated by the cyclooxygenase 2 (COX-2) enzyme (6). This enzyme produces ROS by indirectly activating nicotinamide adenine dinucleotide phosphate (NADPH) oxidase (7). Then, ROS formation leads to renal tubular cell damage by membrane lipid peroxidation, protein dysfunction and DNA breakdown. In addition, ROS formation may indirectly initiate several other pathophysiological processes of IRI such as neutrophil infiltration, endoplasmic reticulum stress and mitochondrial dysfunction (8).

Celecoxib, a member of non-steroidal anti-inflammatory drugs (NSAIDs) family, selectively inhibits COX-2 enzyme activity. There is no report on the increased risk of hematologic side effects and gastrointestinal tract disturbances with the use of NSAIDs (9). Therefore, in order to minimize the damages caused by oxidative stress on the reperfused organs, celecoxib can be a possible choice.

Gender difference in renal IRI has been well established in humans and experimental animals. Females are known to be more resistant to renal IRI than males (10). However, to understand the impact of gender on the effects of celecoxib, this study was designed to compare the protective effects of celecoxib on renal function, oxidative stress status and histopathology in IR-induced AKI between male and female rats with the same degree of injuries.

## Materials and Methods

**Figure 1:**
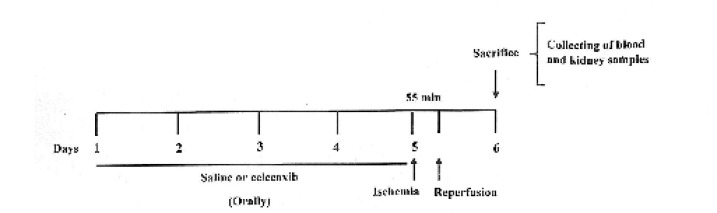
The experimental protocol of study

All experimental procedures (i.e. anesthesia and surgery) were performed according to the standards established by Tehran University of Medical Sciences and the institution’s ethics committee approved the study. Animals were maintained at room temperature (22 ^°^C), 12 hr light-12 hr dark cycle with non-limited 

water and food. Adult male and female Wistar rats were randomly divided into 6 groups (n=6) as previous work in our laboratory: IR, sham and celecoxib groups (11). Male and female rats in the IR and sham groups received saline by gavage for 5 days. Animals in the celecoxib groups were given 30 mg/kg celecoxib orally for 5 days (12). In the fifth day, rats in the IR and celecoxib groups were anesthetized by ketamine (100 mg/kg) and xylazine (10 mg/kg) intraperitoneally (11). Then, they were placed supine and their abdomen was opened through a midline incision. Pedicle of kidneys was exposed and occluded by bulldog clamps bilaterally. After 55 min, renal clamps were released and the kidneys were reperfused for 24 hr. In the sham groups, all of the above processes were performed except the clamping of renal pedicles. At the end of the reperfusion time, the animals were anesthetized and their abdomen was opened. Blood samples were obtained from the inferior vena cava using a 5ml syringe. Then, the samples were centrifuged at 3000 g for 10 min at 4 ^°^C and plasma was collected and stored at -70 ^°^C until analysis for biochemical parameters. Kidneys were also resected and washed in cold phosphate-buffered saline on ice and their capsule was separated. For oxidative stress assays, part of the kidney tissues was frozen in liquid nitrogen quickly and the samples were kept at -70 ^°^C until further study. Other part of the kidney tissues were fixed in %10 formalin for histological assessments (Figure 1).

***Biochemical assays***

Plasma levels of creatinine (Cr) and blood urea nitrogen (BUN) were evaluated as renal functional indices and measured by colorimetric methods using a commercial kit (Pars Azmoon kit) and Hitachi 704 autoanalyser (Hitachi, Tokyo, Japan).

***Measurement of renal oxidative stress indicators***

Malondialdehyde (MDA) levels of renal tissues were determined according to Esterbauer and Cheeseman method. On the basis of this method, MDA reacts with thiobarbituric acid (TBA) (Sigma-Aldrich, USA) and creates a pink pigment, which has a maximum absorbance at 532 nm. The exact amount of MDA in each sample was calculated from the standard curve and presented as μmol/100 mg tissue (13). Renal SOD activities were measured by the method of Paoletti and Mocali. In this assay, superoxide anion is produced from molecular oxygen in the presence of Ethylenediaminetetraacetic acid (EDTA) (Sigma-Aldrich, USA), manganese (II) chloride (Sigma-Aldrich, USA), and mercaptoethanol (Sigma-Aldrich, USA). NADPH oxidation is related to the activation of superoxide anions that are produced in the medium (14). 

***Evaluation of renal histopathology***

Formalin (10% phosphate-buffered) was used to fix the renal tissues. They were then dehydrated by ethanol in different concentrations. Paraffin-embedded renal sections (4 µm) were stained by hematoxylin and eosin. Renal tubules were evaluated based on the extent of the destruction, cellular degeneration and vacuolization, tubular obstruction and formation of luminal debris and casts (11).

***Statistical analysis***

All results are expressed as the mean±SEM. Comparisons between groups were calculated by two-way ANOVA and Tukey’s *post hoc *test. Statistical significance was considered as *P*<0.05.

## Results

***Effect of gender on renal function***

**Figure 2:**
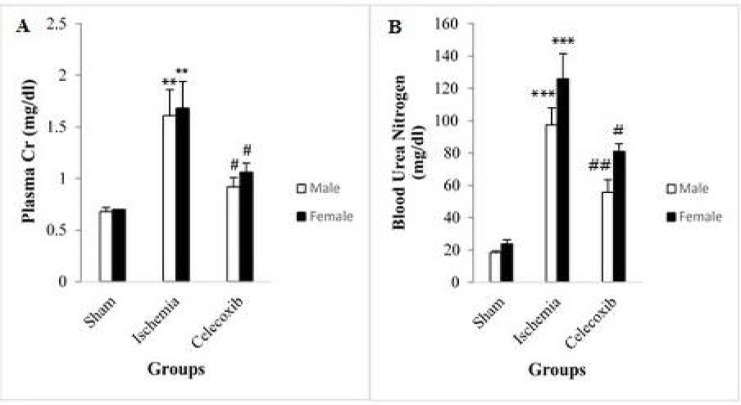
Changes in plasma creatinine (Cr) (A) and blood urea nitrogen (BUN) (B) in different groups. The data are expressed as mean±SEM.

In male rats, plasma Cr level increased significantly in the IR group in comparison with the sham group (1.61±0.25 vs 0.68±0.04 mg/dl, *P*<0.003) (Figure 2A). In female rats, similar results were observed (1.68±0.26 vs. 0.70±0.00 mg/dl, *P*<0.005) (Figure 2A). In male rats, plasma Cr level decreased significantly in the celecoxib group in comparison with the IR group (0.92±0.09 vs. 1.61±0.25 mg/dl, *P*<0.02) (Figure 2A), and similar results were observed in female rats (1.06±0.09 vs. 1.68±0.26 mg/dl, *P*<0.04) (Figure 2A). Plasma Cr level difference was not statistically significant between the male and female groups with the same treatments (Figure 2A).

**P<0.01 versus the related sham group. ***P<0.001 versus the related sham group. #*P<*0.01 versus the related IR group. ##*P<*0.05 versus the male IR group. IR: Ischemia reperfusion

**Figure 3:**
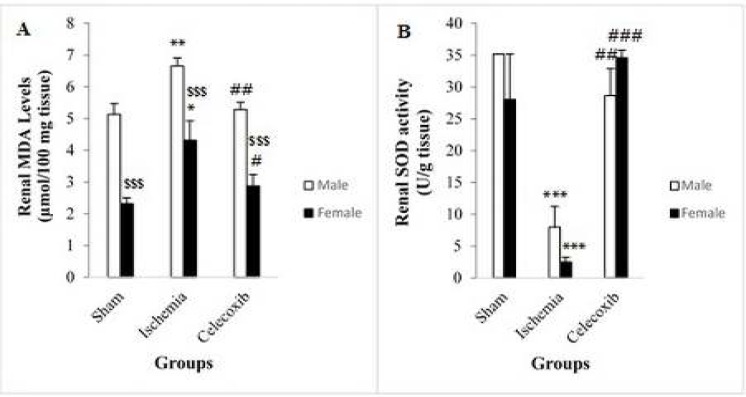
Changes in renal malondialdehyde (MDA) levels (A) and renal superoxide dismutase (SOD) activities (B) in different groups. Data are expressed as mean±SEM. *P<0.05 versus the female sham group. **P<0.01 versus the male sham group. # *P<*0.05 versus the female IR group. ##* P<*0.01 versus the male IR group. ###*P<*0.001 versus the female IR group. $$$*P<*0.001 versus the related male group. IR: Ischemia reperfusion

**Figure 4:**
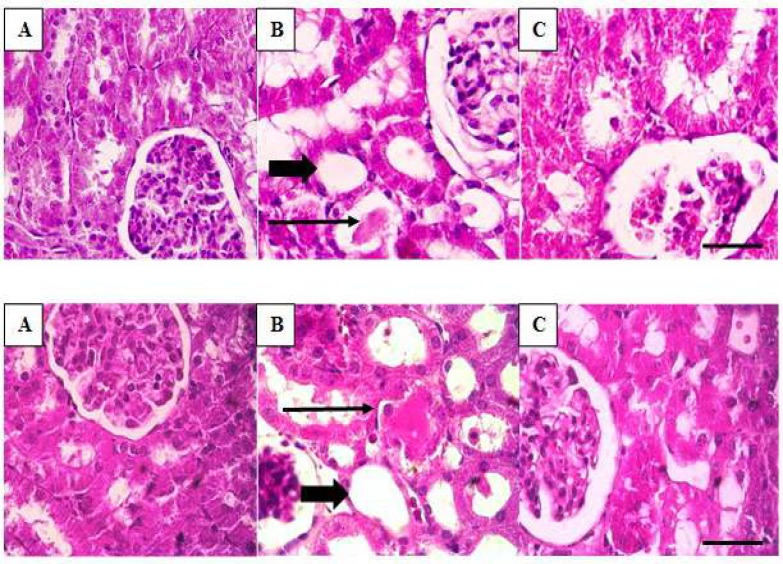
Changes in renal histology in different groups with hematoxylin and eosin staining by light microscopy. Top: (A): sham, (B): IR, (C): celecoxib group in male rats. Bottom: (A): sham, (B): IR, (C): celecoxib groups in female rats. In the rats of the sham groups (A), there was no detectable damage to the renal tissues. In the IR groups (B), some histological changes were observed including disintegration of the tubular cells vacuolation, cast formation and tubular obstruction (long black arrow), flattening of the tubular cells (thick arrow) and necrosis. Administration of celecoxib reduced the extent of these histopathological damages (C). Bar, 100 μm. IR: Ischemia reperfusion

**Figure 5:**
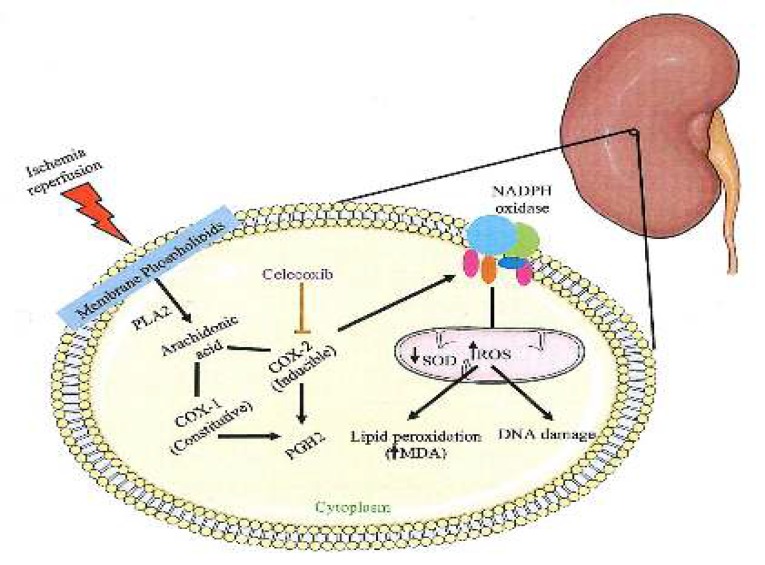
Protective effect of celecoxib against IRI. Celecoxib inhibits COX-2 enzyme activity resulting in the reduction of oxidative stress (ROS formation). PLA 2: Phospholipase A2, COX-1: Cyclooxygenase 1, COX-2: Cyclooxygenase 2, PGH 2: Prostaglandin H2, NADPH: Nicotinamide adenine dinucleotide phosphate, ROS: Reactive oxygen species, SOD: Superoxide dismutase, MDA: Malondialdehyde, DNA: Deoxyribonucleic acid, IRI: Ischemia reperfusion injury

In male rats, plasma BUN level increased significantly in the IR group in comparison with the sham group (97.57±10.33 vs. 18.50±0.92 mg/dl, *P*<0.001) (Figure 2B). In female rats, similar results were observed (126.00±15.44 vs. 23.75±2.59 mg/dl, *P*<0.001) (Figure 2B). In addition, plasma BUN level in male rats decreased significantly in the celecoxib group in comparison with the IR group (55.63±7.91 vs. 97.57±10.33 mg/dl, *P*<0.003) (Figure 2B), and similar results were observed in female rats (81.00±4.70 vs 126.00±15.44 mg/dl, *P*<0.03) (Figure 2B). Plasma BUN level difference was not statistically significant between the male and female groups with the same treatments (Figure 2B).

***Effect of gender on renal oxidative stress status***

In male rats, renal MDA levels increased significantly in the IR group in comparison with the sham group (6.66±0.25 vs. 5.13±0.34 μmol/100 mg tissue, *P*<0.007) (Figure 3A). Similar results were also observed in female rats (4.32±0.61 vs 2.32±0.18 μmol/100 mg tissue, *P*<0.02) (Figure 3A). Renal MDA levels decreased significantly in the male rats of celecoxib group in comparison with the IR group (5.29±0.22 vs 6.66±0.25 μmol/100 mg tissue, *P*<0.01) (Figure 3A). In female rats, similar results were observed (2.88±0.35 vs 4.32±0.61 μmol/100 mg tissue, *P*<0.05) (Figure 3A). However, there were significant differences in renal MDA levels between male and female rats in the sham group (5.13±0.34 vs. 2.32±0.18 μmol/100 mg tissue, *P*<0.001), IR group (6.66±0.25 vs 4.32±0.61, *P*<0.001) (Figure 3A) and celecoxib group (5.29±0.22 vs 2.88±0.35 μmol/100 mg tissue, *P*<0.001) (Figure 3A).

In male rats, renal SOD activities decreased significantly in the IR group in comparison with the sham group (7.96±3.26 vs. 35.18±0.00 U/g tissue, *P*<0.001) (Figure 3B). In female rats, similar results were found (2.50±0.76 vs. 28.06±7.12 U/g tissue, *P*<0.001) (Figure 3B). In male rats, renal SOD activities increased significantly in the celecoxib group in comparison with the IR group (28.66±4.22 vs 7.96±3.26 U/g tissue, *P*<0.003) (Figure 3B), and similar results were reported in female rats (34.59±1.16 vs. 2.50±0.76 U/g tissue, *P*<0.001) (Figure 3B). Renal SOD activity differences were not statistically significant between the male and female groups with the same treatments (Figure 3B).

***Effect of gender on histopathological changes in the kidneys***

In both genders of rats, there was no detectable damage to the kidney tissues of the sham groups (Figure 4A). Renal tissues in the IR groups showed severe changes in the tubules compared to the sham group (Figure 4B). These changes include tubular destruction and disintegration of the tubular cells. Vacuolation, cast formation and tubular obstruction were frequent. Flattening of the tubular cells and necrosis were also observed. By administration of celecoxib, there were reductions in the amount of histopathological damages. In both females and males, tubular destruction and cast formation were reduced (Figure 4C).

## Discussion

In experimental studies, one of the most used models for evaluation of the pathogenesis of renal IRI is occlusion of both renal pedicles (15). Therefore, in the present study, this model was used for induction of renal IRI.

The results of this study demonstrated that IR-induced AKI caused significant increases of plasma Cr and BUN levels and histopathological damages. Renal IR causes extensive alterations in structure and function of the tubular epithelial cells particularly the proximal tubule cells (16). It has been suggested that increased ROS production during renal IR is one of the important causes of renal damage such as extensive interstitial edema, tubular flattening with loss of brush border microvilli, tubular dilation, shedding of brush border, casts and obstruction (17, 18). Another significant event following renal IR is a reduction in the glomerular filtration rate (GFR) that is attributed to decrease of the transglomerular hydraulic pressure gradient as a result of tubular obstruction (19). The reduction of GFR causes impairment of water and electrolyte homeostasis and elevated plasma Cr and BUN levels (i.e. decrease of renal function) (20). Similar to our study, in the study of Liu *et al.*, the IR group showed a significant increase in Cr and BUN levels and extensive morphological abnormalities (21).

However, there were not significant differences between the male and female rats in the IR groups. Several studies have shown that induction of renal IR in females causes less injuries compared to males (22). However, some studies indicated that the required ischemia time to induce IRI in female rats is much more than male rats (e.g. 60 min vs. 30 min) (23, 24). In the present study, the duration of ischemia was well-enough for the establishment of similar injuries in both genders. Therefore, we had an opportunity to investigate the protective effects of celecoxib on both genders with similar degrees of IRI.

Administration of celecoxib significantly prevented renal dysfunction and histopathological damages in IR male rats. Consistent with our results, it was reported that administration of celecoxib attenuated changes of renal functional indicators in a male rat model of cisplatin-induced nephrotoxicity (25). In another study, the authors demonstrated that celecoxib maintained physiological structure of the hepatic tissues in liver ischemia reperfusion in male rats (11). In our study, celecoxib was also capable of significant prevention of renal dysfunction and histopathological damages in female rats. Gender differences of kidneys under physiologic conditions are well demonstrated. For example, two studies independently showed that males have normally higher levels of ROS production compared to females (26, 27). In line with these studies, we also observed an enhanced physiological renal MDA levels in males compared to females in the sham groups.

The exact molecular mechanisms underlying IRI are not fully understood. Nevertheless, several causal factors have been shown to contribute to its pathogenesis. IR causes the liberation of arachidonic acid from membrane phospholipids by phospholipase A2 (PLA 2) enzymes. The released arachidonic acid is metabolized to prostaglandin (PG) H2 by either cyclooxygenase 1 (COX-1) or COX-2 (28, 29). COX-2 enzyme activates NADPH oxidase, which causes oxidative stress (2). In these conditions, it results in excessive production of ROS that is responsible for oxidative damage to biological molecules such as lipids, leading to tissue injury (30).

According to the vital role of COX-2 in renal IRI, several studies have examined the protective effect of selective COX-2 blockade (31). Feiotza *et al*., indicated that COX-2 blockade ameliorated renal damage induced by IRI (32).

Tissue MDA level is a valuable indicator of lipid peroxidation (33). The present study demonstrated that renal MDA levels were significantly higher in the IR group compared to the sham group in both genders. This observation is in accordance with another study that reported an increase in MDA levels in rat kidney after 60 min of ischemia and 15 min of reperfusion (34). However, there were significant differences in renal MDA levels between males and females in the IR groups. Female rats showed less injury compared to the male animals. One explanation for this result is due to the lower renal MDA levels in the sham-related groups. Similarly, it has been reported that renal IR-induced oxidative stress in female was lower than male rats (35).

To remove toxic ROS, cells have several natural defense systems, including SOD enzyme. Increased ROS that is generated during IR may lead to the depletion of these endogenous anti-oxidants (36). In this study, IR-induced AKI significantly caused a decrease of renal SOD activity in both genders. These findings are in good agreement with the study of Sedaghat *et al.* that found reduced SOD activity in renal IR (36).

Celecoxib is a selective inhibitor of COX-2 enzyme and a member of NSAIDs (9).

It prevents downhill pathways of oxidative stress by inhibition of COX-2 enzyme activity (Figure 5). Thus, it is reasonable to assume that celecoxib might provide protection in IRI.

Administration of celecoxib significantly prevented the increase of renal MDA levels and decrease of SOD activities in male rats. Similar to our results, in the study of Ozturk *et al.*, celecoxib decreased MDA levels in the hepatic tissues, and in a study by Koul and Arora, administration of celecoxib augmented the anti-oxidant system in cigarette smoke-induced oxidative stress in mice (12, 37). In this study, celecoxib was also capable of significant suppression of the renal oxidative damages in female rats. Significant improvement of renal MDA levels by celecoxib in female rats may be due to the lower injury in them compared to males.

Limitation of the current study was that the authors did not evaluate the anti-inflammatory effects of celecoxib on both genders in IR-induced AKI. Therefore, we suggest to evaluate the impact of gender on the celecoxib effects on inflammatory indices in IR- induced AKI.

***Clinical implications***

Results of the present study indicate that in conditions such as shock, low cardiac output and kidney transplantation, celecoxib may show protective effects to prevent IR-induced AKI through amelioration of oxidative stress independent of gender.

## Conclusion

In similar pathological conditions, celecoxib improves renal function and histopathological damages and attenuates oxidative damages in both genders by the same degrees independent of gender.
